# The Study on Resolution Factors of LPBF Technology for Manufacturing Superelastic NiTi Endodontic Files

**DOI:** 10.3390/ma15196556

**Published:** 2022-09-21

**Authors:** Stanislav V. Chernyshikhin, Ivan A. Pelevin, Farzad Karimi, Igor V. Shishkovsky

**Affiliations:** 1 Center for Materials Technologies, Skolkovo Institute of Science and Technology, 121205 Moscow, Russia; 2 Catalysis Lab, National University of Science and Technology MISIS, 119049 Moscow, Russia; 3 Department of Materials Science and Engineering, Sharif University of Technology, Tehran 14588 89694, Iran

**Keywords:** selective laser melting, high resolution, μLPBF, laser powder bed fusion, Nickel-Titanium, Nitinol, self-adjusting files

## Abstract

Laser Powder Bed Fusion (LPBF) technology is a new trend in manufacturing complex geometric structures from metals. This technology allows producing topologically optimized parts for aerospace, medical and industrial sectors where a high performance-to-weight ratio is required. Commonly the feature size for such applications is higher than 300–400 microns. However, for several possible applications of LPBF technology, for example, microfluidic devices, stents for coronary vessels, porous filters, dentistry, etc., a significant increase in the resolution is required. This work is aimed to study the resolution factors of LPBF technology for the manufacturing of superelastic instruments for endodontic treatment, namely Self-Adjusting Files (SAF). Samples of thin walls with different incline angles and SAF samples were manufactured from Nickel-Titanium pre-alloyed powder with a 15–45 μm fraction. The printing procedure was done using an LPBF set-up equipped with a conventional ytterbium fiber laser with a nominal laser spot diameter of 55 microns. The results reveal physical, apparatus, and software factors limiting the resolution of the LPBF technology. Additionally, XRD and DSC tests were done to study the effect of single track based scanning mode manufacturing on the phase composition and phase transformation temperatures. Found combination of optimal process parameters including laser power of 100 W, scanning speed of 850 mm/s, and layer thickness of 20 μm was suitable for manufacturing SAF files with the required resolution. The results will be helpful for the production of NiTi micro objects based on periodic structures both by the LPBF and μLPBF methods.

## 1. Introduction

The introduction of rotary instruments into the protocol of mechanical root canal preparation has changed the idea of clinical endodontics. During the irrigation and cleaning procedure, inner walls of the tooth canals undergo an abrasive effect induced by special instruments called files. However, for the proper preparation of the irregular shape canals with conventional files endodontists were obliged significantly expand the canal which result in dentin removal. In 2013, Self-Adjusting File (SAF) made of Nickel-Titanium alloy was introduced, which provided completely new possibilities for minimal invasive endodontic treatments [1,2]. Due to the superelastic properties of the nitinol and the peculiar design of the SAF, the shape of the file adapts to the patient’s tooth canal, which allows the preparation of irregularly shaped canals [2,3]. At the moment, NiTi files are made by laser cutting of hollow tubes. This article explores the possibility of manufacturing SAF using the additive manufacturing (AM) approach.

AM is an indispensable tool for one-step manufacturing of parts with complex geometries from the prescribed 3D CAD model [4]. Laser Powder-Bed Fusion (LPBF), also known as Selective Laser Melting (SLM), is one of the most spread AM technology that allows manufacturing biomimetic and lightweight metallic structures impossible to produce via conventional subtractive technologies [5,6,7]. A distinctive feature of the technology is the principle of subsequent layer-by-layer consolidation of objects and utilization of powder as raw material. The slicer software divides the prescribed 3D model into equal layers and generates trajectories for the laser movement in each slice. After the formation of a homogenous layer of powder on the substrate with a recoater system, a laser beam starts to scan the layer with consistent melting of powder according to the generated hatching zones. The above-mentioned sequence is repeated until the whole part is consolidated.

In terms of comparison between the technologies of traditional laser cutting and LPBF for SAF manufacturing, the following can be stated. The raw material for the LPBF technology can be recycled multiple times which dramatically reduces waste. In paper [8] authors showed that even after the twentieth print the properties of the powder hardly changed and showed high resistance to multiple reuses. The LPBF technology gives rise to a new approach in design through topology optimization [9,10]. The main objective of topology optimization is considered finding the optimal design or shape of a part by minimizing the desired parameter (i.e., mass) with prescribed constraints. Thus, with the AM approach more ideal design of SAF can be built when the design of the final part for laser cutting is constrained by the shape of the blank. Finally, AM provides a straightforward approach to the manufacturing of patient-specific devices [11,12]. However, any research on the manufacturing of NiTi Self-Adjusting Files by LPBF technology was not reported at this moment and cannot be considered an easy task since many requirements are demanded for such application. Resolution factors, an adaptation of SAF design for LPBF, mechanical properties, phase transformation temperatures, and the superelastic response of the material after LPBF, are the main issues for consideration.

The resolution of the LPBF technology and the limiting factors is an important aspect of the technology, and a significant contribution has been made to this topic. In work [13] Wang et al. formulated rules for manufacturing micro porous objects via LPBF. The authors emphasized the importance of taking into account critical inclined angles of overhanging surfaces and adapting a 3D model during slicing to compensate the powder adhesion. The lattice structures with a strut diameter of 300 μm and an inclined angle of 45° were successfully designed and manufactured. Overhanging surfaces with an inclined angle to the substrate plane of less than 45° are known to be the most important geometric restraint in the LPBF technology. In work [14] authors have demonstrated the optimization procedure of the process parameters for overhanging structures. To prevent possible fabricating defects such as warp or fusion of agglomerates to the surface two methods were proposed: adjusting the part in the building volume and controlling energy density near the overhanging surface. However, the influence of the feature size factor on the critical incline level was not revealed. Yadroitsev et al. [15] carry out a study on the technological regimes for filter element manufacturing with a minimum wall thickness of 120 μm from 316L powder. The authors reported results on parametric analysis of the process parameters for manufacturing thin walls from another grade of austenitic stainless steel Inox 904L using the same equipment. The minimum achieved wall thickness was 140 μm and worth to mention that the prescribed 3D model had a 100 μm size. As far as the LPBF resolution is considered, the laser unit and scanning head are known to be the most influential components in the apparatus. Qu et al. [5] reported research on the manufacturing of TPMS structures using a μLPBF machine with a special scanning head. However, the minimum feature size was set not less than 100 μm despite the possible higher resolution. As was shown by Caprio et al. [16] utilization of pulsed lasers provides a smaller melt pool over the same levels of energy density in comparison with CW lasers which leads to an increase in the width of a single track, as a consequence a decrease in the resolution. Wu et al. in paper [17] postulated guidelines on the optimization of the thin wall manufacturing for LPBF. The authors reported experimental data on the minimum wall thicknesses for the Ti6Al4V, AlSi10Mg, and Inconel 718.

Due to the absence of research dedicated to the manufacturing of NiTi Self-Adjusting Files by LPBF technology, it is worth mentioning alternative micro-scale devices that received increased attention. Several works were dedicated to the investigation of the feasibility of printing stents for coronary vessels from Co-Cr [18], titanium [11], steel [19], and NiTi [20], which previously were made using laser cutting methods from hollow tubes [21]. It was demonstrated that resolution, surface finish, and layer bonding dramatically impact the stent performance [18,19,20]. In terms of Nickel-Titanium alloy, both superelasticity and shape memory effect are used for stents to decrease their volume during placement with subsequent extension under temperature influence and to impart a constant load on the walls of the vessel respectively. Furthermore, for intermetallic alloys such as NiTi [22,23,24,25], TiAl [26,27], NiAl [28], etc. application of LPBF technology can remarkably reduce the cost of the parts with complex geometries due to the poor machining of the material associated with high ductility accompanied by a strongly pronounced strain hardening effect [29].

It should be noted that a significant increase in LPBF resolution can be achieved by the utilization of powder with micron or even submicron particles, laser optics with a smaller beam diameter, and thinner layer thickness. The combination of those factors gave rise to a new direction in metal AM, namely Micro Laser Powder Bed Fusion (μLPBF) [30]. However, the emphasis of this work is to study the resolution of standard LPBF equipment with a laser spot size of 55 μm and with the conventional NiTi powder fraction of 15–45 microns. This research will shed light on the main resolution restrictions and approaches for high-resolution 3D printing of thin-walled structures by the LPBF method. Detailed case study on the manufacturing of NiTi endodontic files is performed. The choice of the equipment and research methodology is explained by the augmented possibility to reproduce and use the results obtained in this work for other NiTi micro objects.

## 2. Materials and Methods

The NiTi pre-alloyed powder with Ni content of 55.6 wt.% was produced by NiTiMet Co., Ltd. (Moscow, Russia). The powder was obtained by the electrode induction-melting gas atomization (EIGA) technique. The powder size distribution was analyzed with laser particle sizer Analysette 22 (Fritsch, Idar-Oberstein, Germany). The morphology and chemical composition of particles was studied using Scanning Electron Microscope (SEM) Quattro S (Thermo Fisher Scientific, Waltham, MA, USA). Elemental analysis and mapping of the obtained samples were performed with a Quantax XFlash 6 (Bruker, Billerica, MA, USA) energy dispersive analysis (EDX) attachment. Powder flowability was measured as the time required for 100 g of powder to flow through a standardized 4 mm diameter Hall funnel.

The 3D printing experiments were performed on the AddSol D50 (Additive Solutions, Moscow, Russia) LPBF machine. The installation equipped with an ytterbium fiber laser (IPG Photonics, Fryazino, Russia) yields Gaussian power density distribution, has a nominal laser spot diameter of 55 μm and wavelength of 1064 nm. The building envelope has the form of a cylinder with a diameter of 50 mm and a height of 150 mm. To obtain high-quality bonding between the samples and the substrate in-house-built nitinol base plate was manufactured from NiTi sheet as shown in Section 3.3. For the executive file preparation, the Glicer build processor (version 2.0.1, ATSS solutions, Moscow, Russia) software was used.

**Figure 1:**
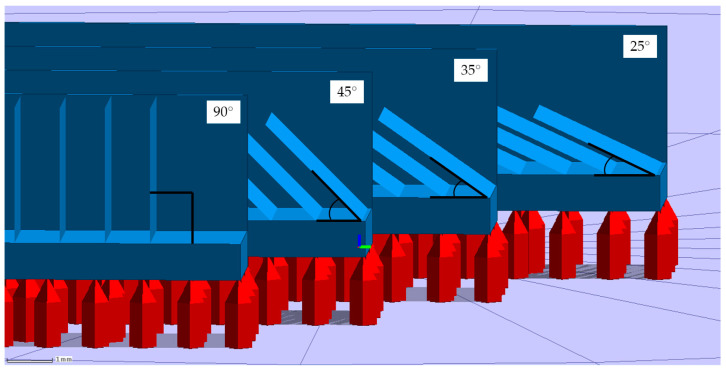
The samples of thin walls in Gliser software.

The first experiment consisted of printing single-track-based thin walls by scanning the layer with one laser passage for a wall per layer. The variables factors of the experiment were laser power and scanning speed with levels presented in Table 1. The layer thickness of 20 μm were considered for all cases. The window of process parameters was chosen according to the previous work [25]. The walls were printed with 4 incline angles: 90°, 45°, 35°, and 25° as shown in Figure 1. The walls were placed on the platform with columnar supports; a stiffener was added to the back to avoid accidental deformation or strains due to relaxation of thermal stresses.

**Figure 2:**
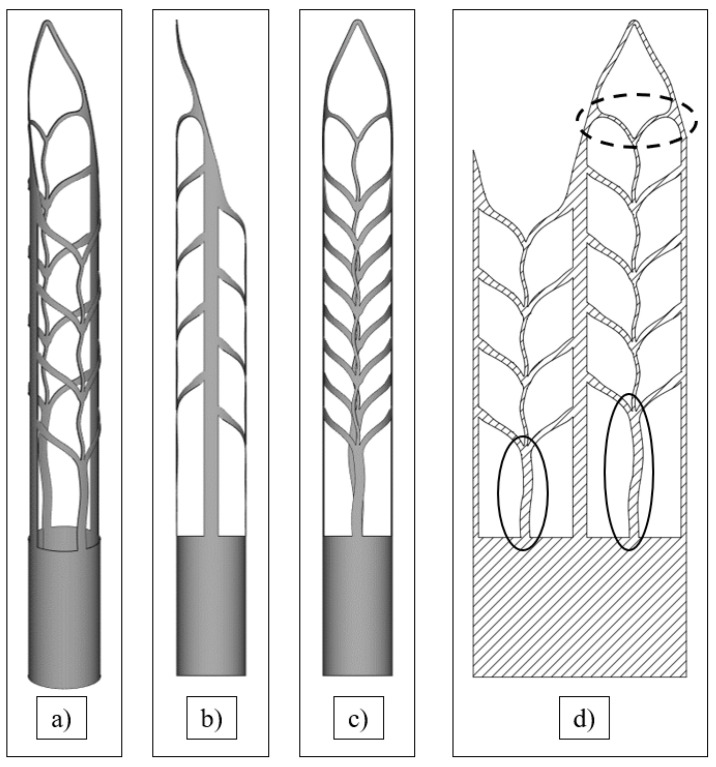
3D-model of Self-Adjusting File adopted for LPBF manufacturing: (**a**) isometry; (**b**) XZ plane view; (**c**) YZ plane view; (**d**) sweep of the SAF, supplemental support strut highlighted with a solid line, critical inclined angle highlighted with a dashed line.

The second experiment consists of 3D printing of SAF files using a narrower window of process parameters. 3D model of Self Adjusted File was designed in Fusion 360 (ver. 2.0.10440, Autodesk, San Francisco, CA, USA). The height of the thin wall structure from the hollow cylinder to the top is 15 mm while the diameter was 2 mm. To manufacture the SAF in a single track-wise manner CAD file with a solid body was converted to *.STL file and then faces with normal vectors pointing inward of the object have been removed to obtain a zero-dimensional thickness. Using this technique, it is possible to achieve the generation of one contour strategy without hatching. 3D-model of SAF is presented in Figure 2a–c. It should be noted that supplemental support struts shown in Figure 2d were added to avoid any removable supports during 3D printing. These struts do not affect the applicability of SAF but simplify the manufacturing procedure significantly.

After the printing procedure, the samples were cut with an electrical discharge machine from the base plate. The thin wall samples were mounter in the epoxy resin, then grinded, and polished. The dimensions of the thin walls were measured with optical microscope AxioScope A1 (Zeiss, Oberkochen, Germany).

The phase transformation temperatures of powder and consolidated sample were determined using Differential Scanning Calorimetry (DSC) analysis with a DSC-60 Plus (Shimadzu, Kyoto, Japan) setup attached to the cooling system. The samples were cut from the struts of the file structure. Cooling/heating rate: 10°/min in the range from −40 °C to 100 °C.

The phase composition was investigated via X-Rad Diffraction (XRD) analysis using the D8 Advance (Bruker, Billerica, MA, USA). The samples were plates cut from the dense part of the file structure. The measurements were carried out at room temperature using an X-ray tube with Cu-Kα radiation (wavelength 0.1504 nm), and Bragg-Brentano focusing. The phase composition of the samples was identified using the 1999 PDF2 X-ray database and BaseDifract (ver. 2.01, Scientific Instruments, Moscow, Russia).

## 3. Results and Discussions

The results of this study are divided into four sections. In the first section, the raw material was comprehensively described. In the second, single track based thin walls were investigated. In the third section, the manufacturing of the SAF files via LPBF technology is reviewed. Finally, in the last section, the XRD and DSC tests were employed to analyze the effect of consolidation on the phase composition and temperatures of the phase transformations.

### 3.1. Raw Powder Characterization

In various works devoted to LPBF technology, it has been shown that the well-chosen initial powder material is the basis for the successful consolidation with minimum defects [31,32,33,34,35]. The most important properties of the powder are particle morphology, chemical composition, and microstructure [31]. In terms of the resolution study, these factors will be fundamental for achieving repeatability. Thus, the initial powder was studied by laser particle sizer, SEM with EDS detector, and Hall funnel.

**Figure 3:**
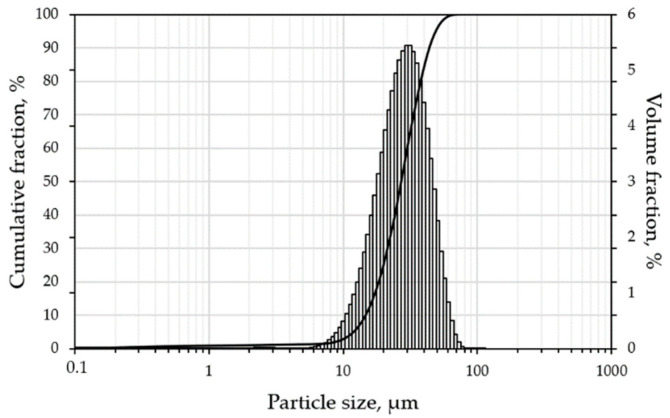
Particle size distribution histogram with cumulative curve.

The results of the granulometric analysis are presented in Figure 3 as a histogram with unimodal normal distribution. The found percentiles of the equivalent diameter are d_10_ = 15.1 µm; d_50_ = 27.8 µm; d_90_ = 45.0 µm. The Particle Size Distribution (PSD) fits the 15–45 µm fraction which is optimal for the LPBF process. The width of the PSD is characterized by the standard deviation near the mean value or SPAN given in the Equation (1):

(1) $$\mathrm{SPAN}=\frac{d_{90}-d_{10}}{d_{50}}=1.076\;$$

For the LPBF process, finer particle content and SPAN value play an important role. Wider PSD will increase theoretical density of the powder as far as finer particles will fill the voids of granular material, however, high content of finer particles will deteriorate flowability due to the formation of agglomerates and increased inter particle friction [36]. During recoating operation the homogeneity of the powder bed is directly affected by the flowability of the powder while inhomogeneity may lead to a lack of fusion defect when regimes with low energy input are applied [37]. Density and consequently thermal conductivity of the powder bed is dependent on the SPAN value [38]. Optimal SPAN value (~1) will result in dense packaging and higher tap density of the powder bed promoting densification without balling effect that occurs due to Plateau-Rayleigh capillary instability [39].

**Figure 4:**
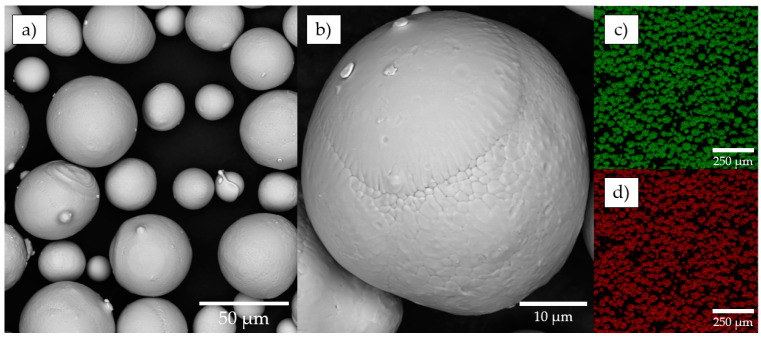
Results of the SEM powder analysis: (**a**) SEM image of powder; (**b**) powder particle with dendritic fragments under high magnification; (**c**) Ni Kα1; (**d**) Ti Kα1.

The morphology and sphericity of the powder particles were characterized using SEM and powder particles shown in Figure 4a. The microstructure of the powder is represented with micro dendrites (see Figure 4b) inherent to the EIGA atomization method. The results of EDS analysis correspond to the Ni-rich chemical composition of the powder (see Figure 4c,d). Found elemental weight ratio of elements is 55.58/44.42 wt.% (Ni/Ti). The difference in chemical composition may lead to a slight shift in the optimal window of process parameters or may affect the functional properties of the final parts due to the evaporation of Ni.

The measured time required to flow 100 g of powder through the Hall funnel was 38.1 ± 1.1 s. Relatively high flowability characteristics of the powder are explained with mean sphericity of 0.91, unimodal PSD, and optimal SPAN. The “satellite” defect represented with smaller particles attached to the surface of the larger ones took place but was not pronounced.

### 3.2. Single Track Based Thin Walls

For optimization of the process parameters for the LPBF process, a two-stage procedure well-established approach is used, consisting of printing single tracks and volumetric samples. Once single tracks were obtained, the hatch distance parameter can be evaluated. At the second step, the mass density is determined for a different combination of laser power and scanning speed with hydrostatic, metallographic, or Micro-CT methods. Finally, for the selection of process parameters, the part design and application should be taken into account. In the following, we propose an approach for optimization of process parameters for the high-resolution LPBF consisting of studying thin walls consolidated in the vertical direction and with different inclined angles.

**Figure 5:**
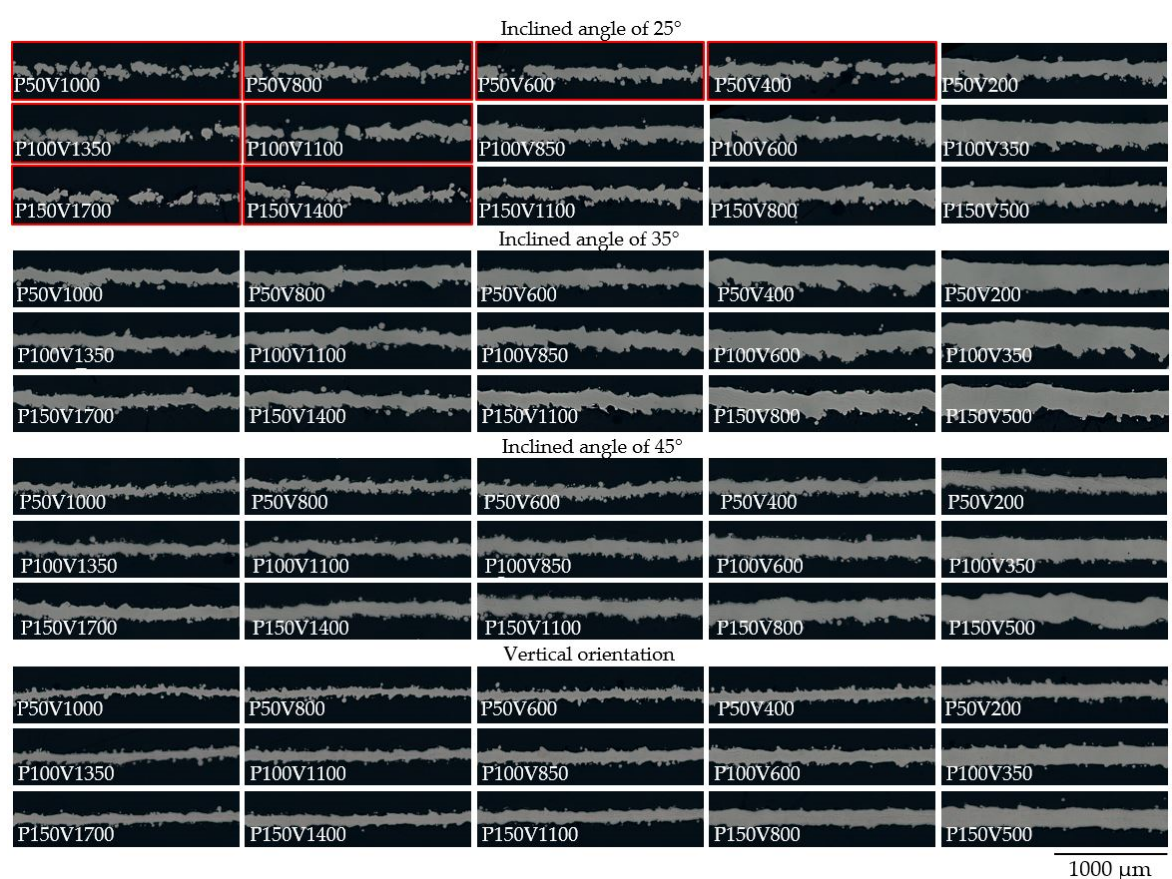
OM of cross-sections for single track based thin walls. Samples with lack of fusion defect are highlighted with red.

Several scanning strategies could be employed for the manufacturing of micro-objects via the LPBF process. The first scanning strategy refers to layer-wise scanning of the powder bed with a single laser passage. The second mode which widely used for the strut-based lattice structures based on the generation of multiple perimeter contours [40] along the border of the 3D model. The third type is the conventional generation of the contours with subsequent hatching. It should be noted that despite the highest resolution achievable with the first mode, most of the build processors and commercial LPBF equipment do not switch to such a scanning strategy when the corresponding feature size of an object is comparable with the width of a single track. Wu et al. [17] found the possibility of switching from the raster mode to the single-bead mode on the EOS equipment. The adopted slicer algorithm used in this study enables gradual change of the scanning mode from the raster to the multiple contours’ generation and finally to the single vectors. In this study, we consider samples produced with the first type of scanning strategy. Optical microscopy (OM) of cross-sections for thin walls consolidated with regimes given in Table 1 are presented in Figure 5. Samples with lack of fusion defect are highlighted with red. The cross-sections are subdivided by the inclined angle to the base plate, as demonstrated in Figure 1, however, all images are presented horizontally.

**Figure 6:**
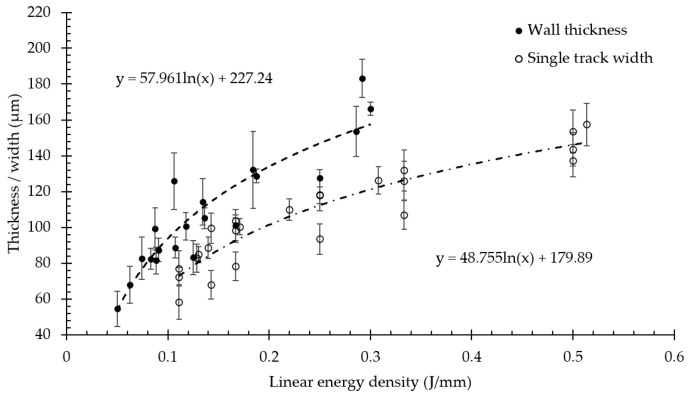
Dependence of wall thickness and width of single tracks [25] on linear energy density for vertical samples.

In Figure 6 experimental measurements of the thicknesses for vertical walls are presented and compared with the width of single tracks adopted from previous work [25]. The minimum wall thickness of 54 ± 10 μm achieved with the linear energy density of 0.05 J/mm is comparable with the laser beam diameter of 55 μm. The corresponding single track width of 58 ± 10 μm was achieved with the energy input of 0.11 J/mm. The width/thickness dependences on the linear energy density demonstrate a consimilar trend and have a logarithmic fit with R^2^ > 0.8. However, the same laser energy input provided greater wall dimensions than the width of the relevant melt pool. Apparently, the phenomenological explanation relates to the difference in the substrates’ properties. For the single track experiment, NiTi base plate was considered as a substrate therefore the heat was evenly distributed from the powder bed to the dense material. For the single track walls, the substrate was the previous layer of thin wall surrounded by powder. It is known that the powder bed has an order of magnitude lower thermal conductivity and half of the density for the bulk material. Thus, the melt pool width may increase due to restrained heat dissipation into the substrate, therefore, augmented heat accumulation by the surrounding powder in case of wall consolidation. Besides the above-mentioned statement, the powder adhesion may increase the average thickness of the wall by the median size of powder particles. The results comply with experimental findings in works [17,41].

**Figure 7:**
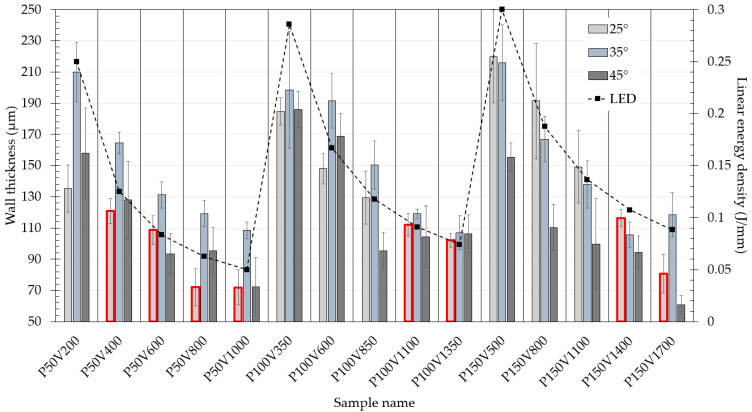
Dependence of wall thickness on linear energy density for inclined walls.

Figure 7 shows the wall thickness with standard deviation measured from samples with inclined angles of 25°, 35°, and 45°. The minimum wall thickness of 61 ± 6 μm was achieved for the P150V1700 sample. For each regime, the linear energy density value is marked with a square marker and dashed line. It is seen that in the case of inclined walls the thickness also correlates with the linear energy density and the minimum value is comparable to vertical specimens. Thus, the minimum wall thickness has a weak dependence on the angle of inclination and is mostly controlled by the melt pool geometry.

However, the linear energy density is hard to use as a parameter for the evaluation of the thickness for several reasons. It was shown in the previous work that the laser power highly affects the linear energy density threshold at which the transition from the conductive mode welding into the keyhole. The linear energy density of 0.30–0.40 J/mm was considered as a threshold based on the experimental melt pool formation analysis and FE simulation results of the temperature fields during LPBF [25]. Therefore, in the present window of technological parameters curves have no singularities commonly associated with rapid changes in the melt pool geometries from the conductive mode welding into a deep keyhole. However, the effect of laser power on the pool stability and formation conditions appears even below the threshold to a lower extent. It is worth mentioning that for the laser power of 50 W the specimens with a 35° inclined angle have a higher value of the wall thickness, for the laser power of 100 W the difference between the thickness of samples with different incline angles is negligible, and for the laser power of 150 W the thickness for the sample with 45° inclined angle have a smaller thickness.

**Figure 8:**
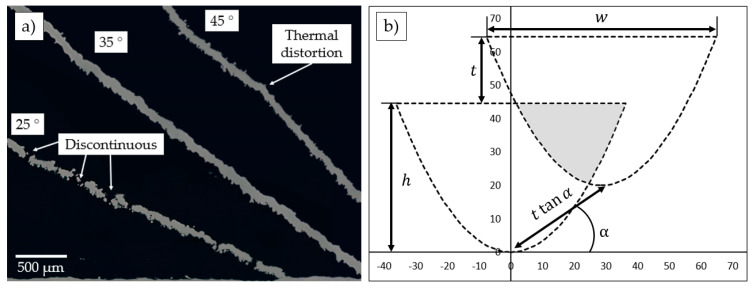
(**a**) OM of walls with different inclined angles; (**b**) schematic represantation of the overlapping beween adjacent tracks during single track manufacturing of inclined objects.

The bars highlighted with red (see Figure 7) represent walls with a lack of fusion defect. Commonly lack of fusion defect is associated with pores filled with unmelted particles and mostly controlled by the overlapping of the adjacent tracks. The lack of fusion defect in the case of single track consolidation leads to the discontinuous structure shown in Figure 8a. Discontinuous element with open pores significantly decreases the mechanical properties of the micro object. The condition of the defect appearance is an interplay among the melt pool size and inclined angle which in turn result in different overlapping of the adjacent tracks. It can be seen that all samples with defects have the lowest inclined angle of 25° and low linear energy density. A simple model can be used to evaluate the limits of both factors. Two adjacent layers are considered in the perpendicular plane to the laser scanning vector. In each layer a melt pool represented with parabolic shape and overlapping is estimated. We consider following assumptions: the shape of the melt pool corresponds to the shape obtained during the scanning of the powder bed on the bulk substrate, the melt pool upper surface is flat, the formation of the tracks is stable. As input parameters, layer thickness (*t*), inclined angle (α), height (h), and width (w) of the melt pool are considered. The dimensions in Figure 8b represent the experimentally measured data on the single tracks for the P150V1400 from [25]. In this case, (ttanα) parameter can be used for the single track consolidation as an analog of hatch distance. Accordingly, for samples P150V1400, P150V1100, and P150V800 calculated overlapping areas are 8.8, 17.7, and 29.7% respectively. In prescribed process parameters matrix samples with overlapping areas below 10% resulted in a lack of fusion defect. However, more experimental data on the melt pool geometry is required to formulate limitations for inclined object manufacturing based on this model.

### 3.3. High Resolution Fabrication of Self-Adjusting Files

**Figure 9:**
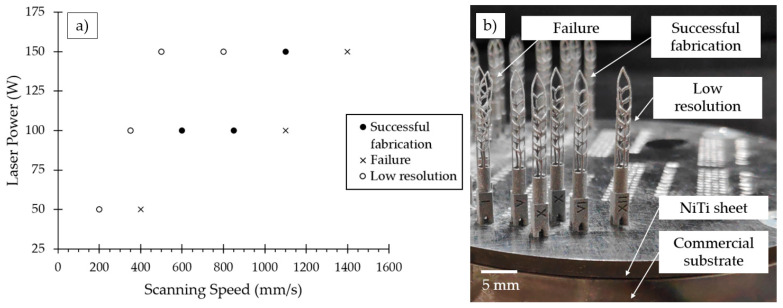
(**a**) Matrix of LPBF regimes of Self-Adjusting files fabrication; (**b**) Produced samples of SAF.

The technological parameters for SAF samples manufacturing were determined based on the results of Section 3.2. The corresponding matrix in the laser power and scanning speed coordinates is presented in Figure 9a. All regimes are subdivided into successful fabrication, failure, and low resolution. The SAF samples on the NiTi substrate after the LPBF consolidation are presented in Figure 9b. Samples P50V400, P100V1100, and P150V1400 were highly distorted and disconnected in the most inclined struts marked in Figure 2d with a dashed line. The inclined angle of the struts reaches a minimum of 24° in this place. This results in a good agreement with the preliminary study of the single track thin walls.

**Figure 10:**
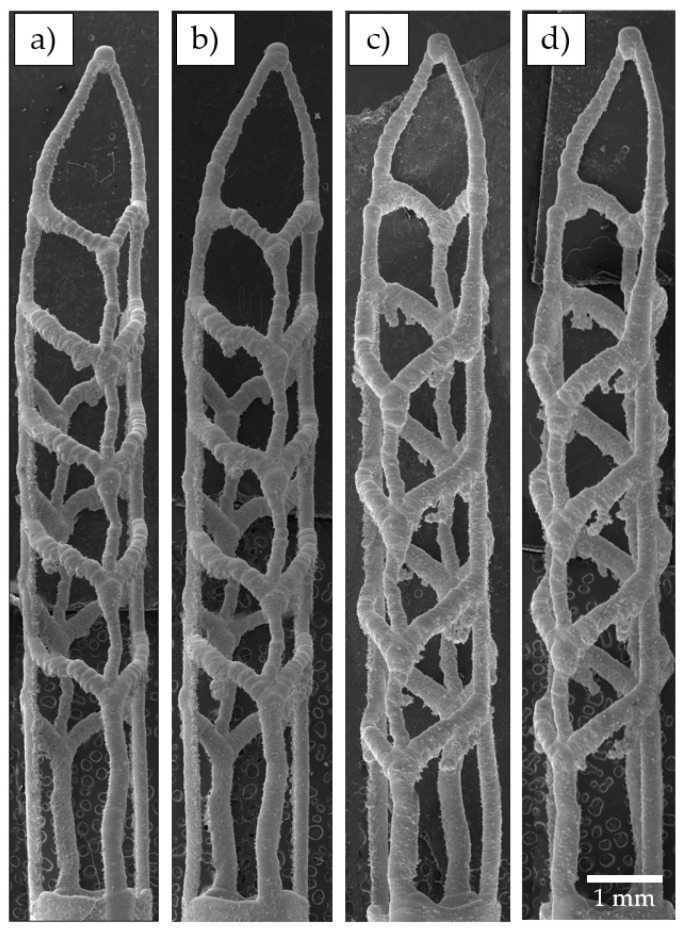
3D-printed SAF samples fabricated with various regimes: (**a**) P100V850, (**b**) P150V1100, (**c**) P100V400, (**d**) P50V200.

SEM images of 3D-printed SAFs manufactured with regimes P100V850, P150V1100, P100V400, and P50V200 are shown in Figure 10. As a criterion for the low-resolution regimes, SAF samples with a strut diameter over 150 μm in the upper part were considered. It should be noted that with the minimum laser power of 50 W, the desirable resolution was not achieved despite resemblant values of linear energy density for other cases. Such a trend can be related to reduced scanning speed or increased melt pool lifetime. The kinetics of powder denudation and particles retraction into the melt pool will be highly affected. Agglomerates fused to the struts of the SAF samples can be seen in Figure 10c,d the appeared within the low scanning speed regimes.

**Figure 11:**
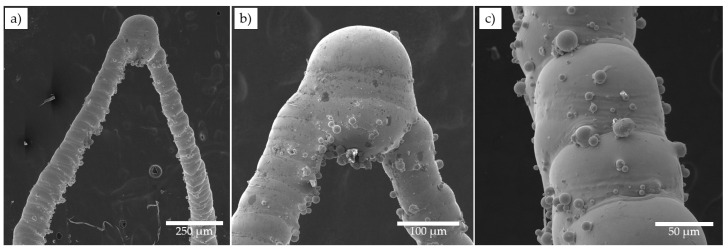
High magnification images of 3D-printed SAF sample P100V850: (**a**) the upper part, (**b**) the tip, (**c**) the strut of upper part.

In Figure 11 SEM images of the upper part of the P100V850 SAF sample is presented at different magnifications. The tip region is shown in Figure 11a,b accumulated the highest deviations from the prescribed single track design. There is no thickening of the tip in the 3D model (see Figure 2a), but it occurs on the printed sample and arises because of quasi-continuous laser mode which does not allow to make a single pulse of laser energy in the point. However, such thickening does not affect SAF performance (abrasive cleaning). The inherent layering effect can be clearly observed in Figure 11c. Since the SAFs were manufactured on the edge of resolution such layering effect is inevitable. Besides, fused particles on the SAF surface are presented that are common surface defects [20], but considering synthesized object dimensions these defects become more influential. Both surface irregularities (layering and fused particles) possibly can be removed by chemical etching [42].

### 3.4. Characterization of SAF Sample Made with Optimal Process Parameters

**Figure 12:**
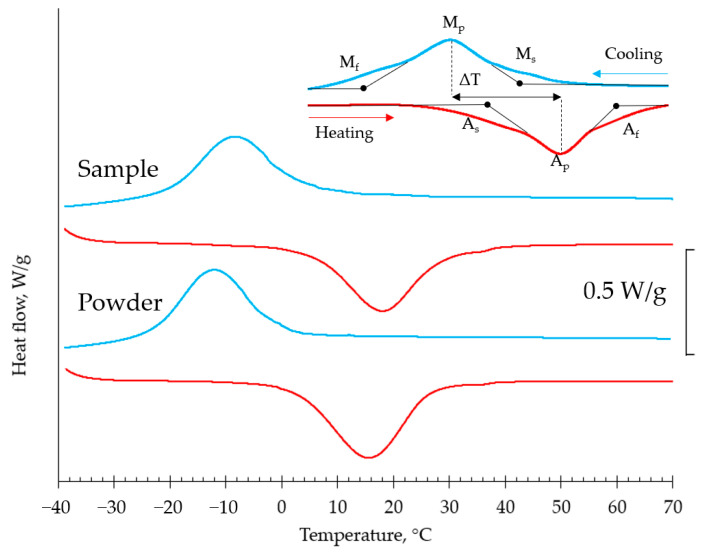
Differential scanning calorimetry for raw powder and sample P100V850.

As-built P100V850 sample that was found to be optimal for manufacturing of single track SAF files were subjected to DSC testing and compared with the raw powder DSC results. Figure 12 shows the typical DSC curves for raw powder and a cut sample from a file. The exact values of characteristic transformation temperatures were found for reversible martensite phase transition during the heating/cooling cycle and presented in Table 2. The DSC curves are represented with a single peak that corresponds to the direct cubic-monoclinic B2-B19′ transformation without intermediate rhombohedral R phase. It is seen from Figure 12 that thermal hysteresis of the transition is observed which is typical for NiTi alloys [43].

For the enhanced superelastic response of the NiTi parts, Af temperature of the transformation should be below the working temperature by 10 °C [44]. Measured transformation temperatures presented in Table 2 indicate that characteristic temperatures were increased after the LPBF procedure, especially Ms (from −1.1 to 3.8 °C). As far as martensite transformation is sensitive to Ni-content, this shift could occur due to Ni evaporation during laser influence. It was demonstrated in the paper [45] that the hatch distance parameter has the highest influence on the resulting transformation temperatures by means of grain size and orientation. A decrease in the hatch distance led to the finest melt pools due to multiple remelting and intense overlapping. Apparently, the single track strategy manufacturing, on the contrary, may have the lowest impact which is also indicated by the obtained experimental data. Thus, achieving enhanced superelastic response can be simplified in the micro objects produced via LPBF by the prescribed approach.

**Figure 13:**
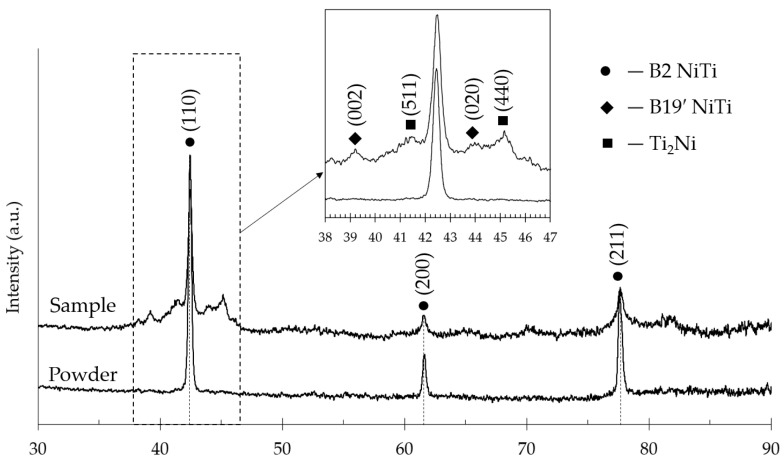
XRD patterns of initial NiTi powder and 3D-printed P100V850 sample.

The X-ray diffractograms recorded at room temperature of raw powder and as-build file are presented in Figure 13. It is seen that the initial phase composition of the powder was represented by the high-temperature B2 austenite phase. After the LBPF process, a few peaks of a low-temperature phase (B19′-martensite) were observed which align well with the found increase of the A_f_ temperature in the DSC analysis. However, both diffractograms primarily correspond to B2-phase with typical X-ray peak intensity ratio close to isotropic NiTi alloy: very strong {110}, weak {200} and medium-strong {211}.

Despite the amount of martensite phase in the as-built state does not exceed 5% this may lead to irreversible deformations during the loading/unloading loop in the superelastic region. Additionally, small peaks of the Ti_2_Ni phase were observed in the low-angle region. The formation of the Ti_2_Ni-phase leads to depletion of the solid solution in Ti and a corresponding decrease in the martensitic transformation temperatures (Figure 12 and Table 2). Thus, the shift of transformation temperatures is a result of several opposite processes’ interaction, i.e., Ni evaporation and Ti-rich phase formation.

## 4. Conclusions

In the present work, general approaches have been developed for high-resolution 3D printing of thin-walled structures by the LPBF method. The main resolution restrictions were discussed and a detailed case study on the manufacturing of NiTi endodontic files was performed. Based on the obtained results following conclusions were formulated:

(1) Within this work the feasibility of manufacturing endodontic Self-Adjusted Files from Nickel-Titanium shape memory alloy was demonstrated via conventional LPBF technology with found optimal process parameters including laser power of 100 W, scanning speed of 850 mm/s, and layer thickness of 20 μm.
(2) The key factor limiting the resolution of LPBF technology is the melt pool dimensions; if the technological window for raw material is wide enough, the combination resulting in the smallest width and depth of the melt pool will be required for the manufacturing of objects with small feature size, i.e., endodontic files, strut-based structures, coronary stents, porous micro-implants, etc. The minimum wall thickness of 54 ± 10 μm was achieved for the NiTi powder which is close to the laser beam diameter.
(3) Accuracy and resolution of final parts can be increased by special algorithms to slicing software for implementation of scanning strategy based on single vectors without contours and subsequent filling of hatch lines. In single track based manufacturing inclined angle of the part plays an important role. It was demonstrated that the critical value for micro objects is dependent on layer thickness and linear energy density. Successful fabrication of micro object with an inclined angle of 24° was demonstrated which is significantly lower than the conventional limit of 45°.
(4) Powder adhesion and the layering effect inherent to LPBF technology have a higher impact on the manufacturing of the micro object with high resolution. Such surface irregularities are inevitable; however, post-treatment can be applied to decrease the roughness of the part elements and the diameter of the struts.
(5) The martensite phase transformation of the SAF sample produced via LPBF was shifted to higher temperatures in comparison with the powder. Such change can be attributed to the Ni evaporation and formation of the Ti_2_Ni phase that was detected by the XRD analysis.

Future studies will be conducted regarding the superelastic properties of the printed parts, fatigue properties in cyclic tests, and preclinical trials. The developed approach can be implemented for other materials and complex lattice (hierarchical) structures to use in 3D printing by the LPBF method.

## 5. Patents

The authors S.C. and I.S. declare that a portion of the content presented in this paper has been applied as a patent for an invention. The application has been submitted to the Russian Federal Institute of Industrial Property with the following details:

Title: Method for Direct Laser Synthesis of Superelastic Endodontic Instruments from Nickel-Titanium alloy.

Application number: 2022117224 (Submission date: 22 June 2022).

## Figures and Tables

**Table 1:** The window of process parameters used for the optimization procedure.

Sample Name	Laser Power, W	Scanning Speed, mm/s	Linear Energy Density, J/mm
P50V200	50	200	0.25
P50V400	50	400	0.13
P50V600	50	600	0.08
P50V800	50	800	0.06
P50V1000	50	1000	0.05
P100V350	100	350	0.29
P100V600	100	600	0.17
P100V850	100	850	0.12
P100V1100	100	1100	0.09
P100V1350	100	1350	0.07
P150V500	150	500	0.30
P150V800	150	800	0.19
P150V1100	150	1100	0.14
P150V1400	150	1400	0.11
P150V1700	150	1700	0.09

**Table 2:** Temperatures of phase martensitic transition of initial powder material and fabricated sample.

Sample Name	Transformation Temperatures (°C)						
	M_s_	M_p_	M_f_	A_s_	A_p_	A_f_	ΔT
NiTi raw powder	−1.1	−12.2	−22.8	2.5	15.5	26.3	27.7
P100V850 sample	3.8	−8.5	−20.2	4.7	18.3	29.7	26.8

## Data Availability

Data sharing is not applicable for this article.
